# Effect of Antioxidant Vitamin Supplementation on Cardiovascular Outcomes: A Meta-Analysis of Randomized Controlled Trials

**DOI:** 10.1371/journal.pone.0056803

**Published:** 2013-02-20

**Authors:** Yizhou Ye, Jing Li, Zhongxiang Yuan

**Affiliations:** 1 Department of Cardiovascular Surgery, Shanghai First People's Hospital, Shanghai Jiaotong University, Shanghai, China; 2 Department of Cardiology, Children's Hospital of Fudan University, Shanghai, China; Universidad Peruana de Ciencias Aplicadas (UPC), Peru

## Abstract

**Background:**

Antioxidant vitamin (vitamin E, beta-carotene, and vitamin C) are widely used for preventing major cardiovascular outcomes. However, the effect of antioxidant vitamin on cardiovascular events remains unclear.

**Methodology and Principal Findings:**

We searched PubMed, EmBase, the Cochrane Central Register of Controlled Trials, and the proceedings of major conferences for relevant literature. Eligible studies were randomized controlled trials that reported on the effects of antioxidant vitamin on cardiovascular outcomes as compared to placebo. Outcomes analyzed were major cardiovascular events, myocardial infarction, stroke, cardiac death, total death, and any possible adverse events. We used the I^2^ statistic to measure heterogeneity between trials and calculated risk estimates for cardiovascular outcomes with random-effect meta-analysis. Independent extraction was performed by two reviewers and consensus was reached. Of 293 identified studies, we included 15 trials reporting data on 188209 participants. These studies reported 12749 major cardiovascular events, 6699 myocardial infarction, 3749 strokes, 14122 total death, and 5980 cardiac deaths. Overall, antioxidant vitamin supplementation as compared to placebo had no effect on major cardiovascular events (RR, 1.00; 95%CI, 0.96–1.03), myocardial infarction (RR, 0.98; 95%CI, 0.92–1.04), stroke (RR, 0.99; 95%CI, 0.93–1.05), total death (RR, 1.03; 95%CI, 0.98–1.07), cardiac death (RR, 1.02; 95%CI, 0.97–1.07), revascularization (RR, 1.00; 95%CI, 0.95–1.05), total CHD (RR, 0.96; 95%CI, 0.87–1.05), angina (RR, 0.98; 95%CI, 0.90–1.07), and congestive heart failure (RR, 1.07; 95%CI, 0.96 to 1.19).

**Conclusion/Significance:**

Antioxidant vitamin supplementation has no effect on the incidence of major cardiovascular events, myocardial infarction, stroke, total death, and cardiac death.

## Introduction

Cardiovascular disease is the leading cause of premature morbidity and mortality worldwide for both men and women [1]–[2]. Over the past few decades, many studies have shown that LDL-cholesterol may be rendered more atherogenic by oxidative modification that allows it to accumulate in the artery walls, and antioxidants have been shown to slow the progression of atherosclerosis [3]–[5]. Therefore, it has been suggested that raised concentrations of antioxidant vitamin in the blood should be as a therapeutic approach to prevent cardiovascular disease [6]–[8]. However, a raised concentrations of antioxidant vitamin in the blood has not consistently been shown to be beneficial [8]–[9].

Antioxidant vitamins (vitamin E, beta-carotene, and vitamin C) are clearly effective at raising concentration of plasma antioxidant vitamin [10]–[11]. Vitamin E and Beta-carotene could substantially prolong the resistance of LDL particles to oxidative damage, and have other potentially protective effects [10]. Vitamin C is a major water-soluble antioxidant in the plasma, and it can help to regenerate oxidized vitamin E. Most randomized controlled trials evaluated the effects of combination of vitamins on the risk of major cardiovascular outcomes. However, their effects on cardiovascular outcomes remain unclear. In addition, previous trials [9], [12] have shown that elevating the plasma antioxidant vitamin does not improve cardiovascular outcomes, previous meta-analysis [13] also failed to show the benefit of antioxidant vitamin supplementation for diverse populations. which makes interpretation of the results difficult for clinicians and has further restricted its application in clinical prevention.

Recently, several large-scale randomized controlled trials investigating antioxidant vitamin supplementation have been completed [9], [12], [14], [15]. For a better understanding of the effect of antioxidant vitamin supplementation on cardiovascular outcomes, data from these recent trials need to be assessed to formulate a conclusion. We therefore did a systematic review and meta-analysis of pooled data from randomized controlled trials, which including the latest evidence of the association of antioxidant vitamin supplementation on the risk of cardiovascular outcomes and any possible adverse events.

## Methods

### Literature Search

We gathered data from randomized controlled trials to assess the effect of antioxidant vitamin supplementation on the risk of cardiovascular outcomes. Trials reported in English were eligible for inclusion, regardless of publication status (published, unpublished, in press, or in progress). Relevant trials were identified with the following procedure:

1. Electronic searches: we searched PubMed, EmBase, and the Cochrane Central Register of Controlled Trials for articles from Jan, 1970 to June 20, 2012. We used the terms of ‘tocopherol’, ‘ascorbic acid’, ‘β-Carotene’, ‘cardiovascular disease’, ‘coronary disease’, ‘coronary thrombosis’, ‘ischemic heart disease’, ‘stroke’, ‘coronary stenosis’, ‘coronary restenosis’, ‘human’, ‘English’, and ‘randomized controlled trials’.
2. Other sources: we contacted authors to obtain any possible additional published or unpublished data. Furthermore, we searched ongoing trials in the metaRegister of Controlled Trials, which lists trials that are registered as completed but not yet published. In addition, we reviewed bibliographies of achieved publications for potentially relevant articles. Finally, we searched in http://www.who.int/trialsearch and http://www.ClinicalTrials.gov websites for information on registered randomized controlled trials. This review was conducted and reported according to the PRISMA (Preferred Reporting Items for Systematic Reviews and Meta-Analysis) Statement issued in 2009 (Table S1) [16].

### Selection Criteria

The literature search was undertaken independently by 2 authors with a standardized approach. Any inconsistent between these 2 reviewers was settled by group discussion until a consensus was reached. We restricted our research based on randomized controlled trials, which contributed less confounding and bias than based on observational studies. All completed trials evaluating the effects of antioxidant vitamin on cardiovascular outcomes as compared to placebo, and providing at least 1 outcome as follows: major cardiovascular events, myocardial infarction, stroke, total death, or cardiac death.

### Data Collection and Quality Assessment

Data abstraction and quality assessment were conducted independently by 2 reviewers using a standardized approach. Any discrepancy was adjudicated by a third reviewer after referring to the original articles, and then the primary author made the final decision. Extracted information included first author or study group’s name, the year of publication, the number of patients enrolled, mean age, percentage male, history of disease, intervention, the duration of follow-up, and outcome events. Disagreements regarding the data were settled by consensus between all authors. The quantitative 5-point Jadad score [17] was used to evaluate the quality of the inclusive trials based on randomization, concealment of the treatment allocation, blinding, completeness of follow-up and the use of intention-to-treat analysis.

### Statistical Analysis

We allocated the results of each randomized controlled trial as dichotomous frequency data. Individual relative risks (RRs) and 95% confidence intervals (CIs) were calculated from event numbers extracted from each trial before data pooling. The overall relative risk (RR) with its 95% CIs of serious cardiovascular events and any possible adverse events were also calculated. Furthermore, we also conducted a subgroup analyses based on publication year, number of patients enrolled, mean age, sex, history of disease, the duration of follow-up, and Jadad score to assess the effect of antioxidant vitamin on cardiovascular outcomes in some specific population. The statistical estimates of effect were derived using a random-effect model with Mantel-Haenzel statistics [18]–[19]. Heterogeneity of treatment effects among included trials was investigated visually by scatter plot analysis and statistically by the heterogeneity I^2^ statistic [20]–[21]. According to Cochrane criteria [21], I^2^ statistic of 0%–40% indicates unimportant heterogeneity, 30%–60% indicates moderate heterogeneity, 50%–90% indicates substantial heterogeneity, and 75%–100% indicates considerable heterogeneity. P values were calculated by x^2^ tests. We also evaluated the probability of publication bias with Egger’s test [22] and Begg-Mazumdar test [23]. All the reported P values were two-sided and statistical significance was defined as a P value less than 0.05. All analyses were calculated using STATA software (version 10.0).

## Results

### Search Of The Published Literature

**Figure 1:**
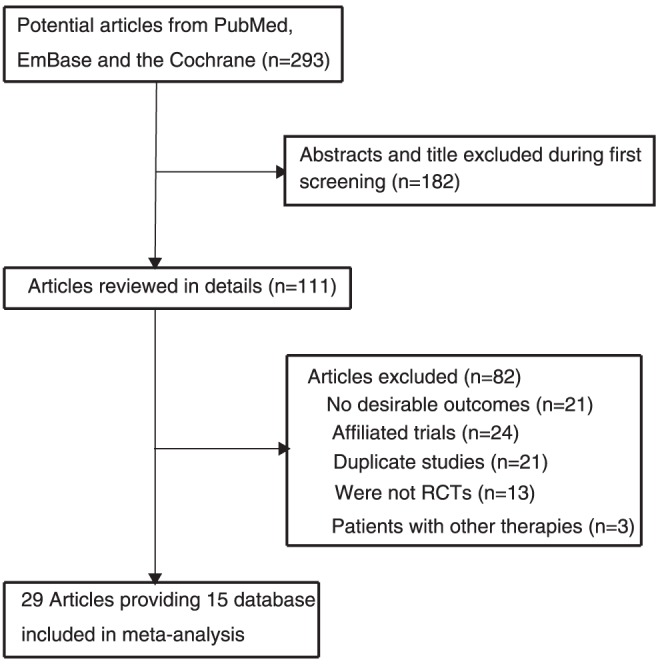
Flow diagram of the literature search and trial selection process.

We identified 293 articles from our initial electronic search, of which 234 were excluded during a preliminary review (title and abstract), we retrieved the full text of the remained 59 studies, and 29 study [9], [12], [14], [15], [24]–[48] providing fifteen database met the inclusion criteria (Figure 1 and Figure S1 [16]), which consisted of data of 188209 individuals.

### Characteristics Of The Included Studies

Table 1 summarized the baseline characteristics of the included studies and their participants. Of these, seven trials [15], [29], [41], [42], [44], [47], [48] evaluated antioxidant vitamin supplementation in participants with high cardiovascular risk factors or previous cardiovascular disease, and the remaining eight trials reported individuals without high cardiovascular risk factors [9], [12], [14], [24]–[28], [30]–[40], [43], [45], [46]. The number of patients included in every study ranged from 80 to 39876, mean age of included trials for participants ranged from 49 to 66, and the follow-up for participants ranged from 1.4 to 12 years. Eight [9], [12], [14], [15], [24], [25], [32]–[42], [44] of included trials evaluated combination of vitamins compared with placebo, and the remaining seven trials [26]–[31], [43], [45]–[48] evaluated single antioxidant vitamin compared with placebo. The outcomes shown major cardiovascular events were available in 12 trials, myocardial infarction in 12 trials, stroke in 10 trials, death from any course in 13 trials, cardiac death in 12 trials, revascularization in 4 trials, total coronary heart disease (CHD) in 2 trials, angina in 2 trials, and congestive heart failure in 3 trials. Jadad score was used to evaluate the quality of the included trials. Overall, two trial had a Jadad score of 5, seven scored 4, four scored 3, and remaining two scored 2.

**Table 1:** Design and characteristic of trials included in our meta-analysis.

Source	No. of patients	Sex (male,%)	Mean age, y	Subjects	Intervention	Follow-up (year)	Jadad score
The SU.VI.MAX Study (2004) [24]–[25]	13017	39	49	Volunteers	Combination of 120 mg of ascorbic acid, 30 mg of vitamin E, 6 mg of beta carotene, 100 µg of selenium, and 20 mg of zinc; placebo	7.5	4
WAC Study (2007) [9], [12]	8171	0	61	Health professionals	Ascorbic acid (500 mg/d), vitamin E (600 IU every other day), and beta carotene (50 mg every other day); placebo	9.4	4
The PHS II Study (2008) [14]	14671	100	64	754 men (5.1%) with cardiovascular disease	400 IU of vitamin E every other day and 500 mg of vitamin C daily; placebo	8	5
The WHS Study (2005) [26]–[28]	39876	0	55	Health professionals	600 IU of natural-source vitamin E on alternate days; placebo	10.1	5
The HOPE and HOPE-TOO Trial (2005) [29]	9541	73.	66	High risk for cardiovascular events	Daily dose of natural source vitamin E (400 IU); placebo	7.0	4
CARET Study (1996) [30]–[31]	17140	66	58	Asbestos-related lung disease or have worked in specified high-risk trades	Combination of 30 mg of beta carotene per day and 25,000 IU of vitamin A per day; placebo	4.0	3
ATBC Cancer Prevention Study (1998) [32]–[40]	27271	100	57	Smoker	Vitamin E (50mg), beta-carotene (20mg), or both above; placebo	6.1	3
CLIPS Group (2007) [15]	366	71	66	Peripheral arterial disease	600 mg vitamin E, 250 mg vitamin C and 20 mg b-carotene daily; placebo	2	3
DD Waters (2002) [41]	423	0	65	Postmenopausal women with at least one 15% to 75% coronary stenosis	400 IU of vitamin E twice daily plus 500 mg of vitamin C twice daily; placebo	2.8	4
BG Brown (2001) [42]	80	87	53	Coronary disease, low HDL, and normal LDL	800 IU of vitamin E, 1000 mg of vitamin C, 25 mg of natural beta carotene, and 100 µg of selenium; placebo	3.2	4
ER Greenberg (1996) [43]	1720	69	63.2	Basal cell or squamous cell skin cancer treated	50 mg of beta carotene; placebo	8.2	2
HPSC Group (2002) [44]	20536	NG	40–80	Coronary disease, other occlusive arterial disease, or diabetes	600 mg vitamin E, 250 mg vitamin C, and 20 mg beta-carotene; placebo	5	4
The PHS Study (1996) [45]–[46]	22071	100	40–84	11% were current smokers and 39% were former smokers	Beta carotene 50 mg on alternate days; placebo	12	3
The CHAOS Investigators (1996) [47]	2002	84	62	With angiographically proven coronary atherosclerosis	Vitamin E 400 or 800 IU daily; placebo	1.4	2
GISSI-Prevenzione Investigators (1999) [48]	11324	85	59	Previous with myocardial infarction	Vitamin E 300 mg daily; placebo	3.5	4

### Effects of Interventions

**Figure 2:**
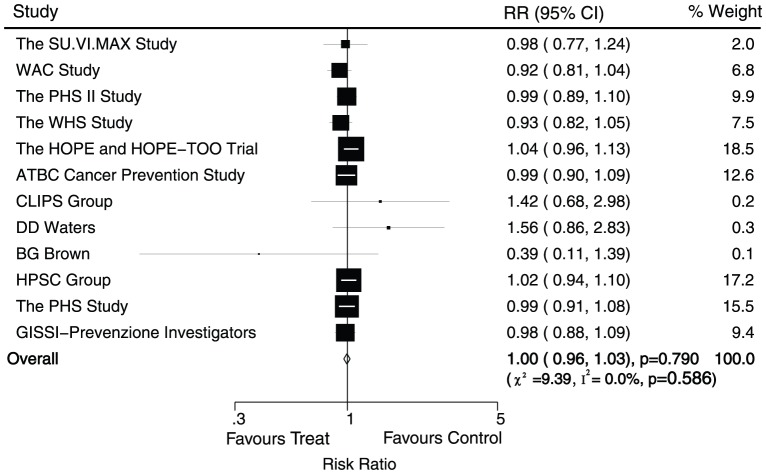
Effects of antioxidant vitamin supplementation on the risk of major cardiovascular events.

Data for the effect of antioxidant vitamin supplementation on major cardiovascular events were available from 12 trials, which included 167051 individuals and reported 12749 serious vascular events. Figure 2 shows the effect of antioxidant vitamin supplementation on major cardiovascular events when compared with placebo, we noted that antioxidant vitamin supplementation does not effect on major cardiovascular events (RR, 1.00; 95%CI, 0.96–1.03; P = 0.79, without evidence of heterogeneity of effect, Figure 2).

**Figure 3:**
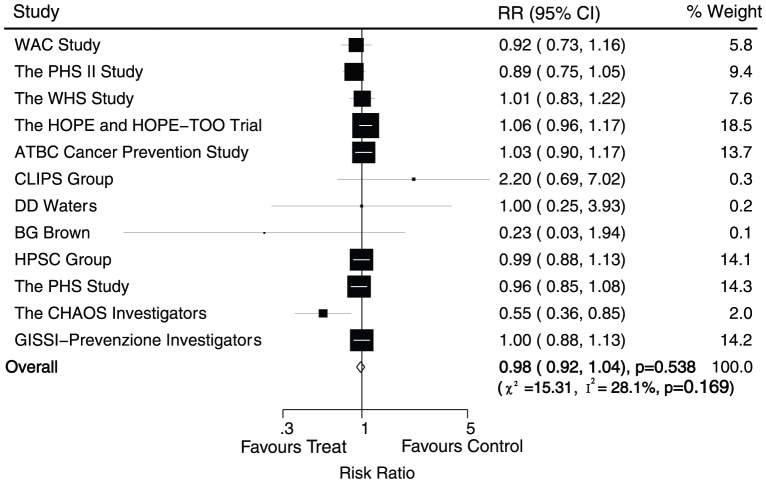
Effects of antioxidant vitamin supplementation on the risk of myocardial infarction.

Data for the effect of antioxidant vitamin supplementation on myocardial infarction were available from 12 trials, including 156312 individuals with 6699 myocardial infarction. Overall, we noted that antioxidant vitamin supplementation reduced the risk of myocardial infarction by 2%, but this difference was not associated with a clinically and statistically significant (RR, 0.98; 95%CI, 0.92–1.04; P = 0.54, with unimportant heterogeneity, Figure 3). Although there was some evidence of heterogeneity across the trials included, a sensitivity analysis indicated that the results were not affected by sequential exclusion of any particular trial from all pooled analysis.

**Figure 4:**
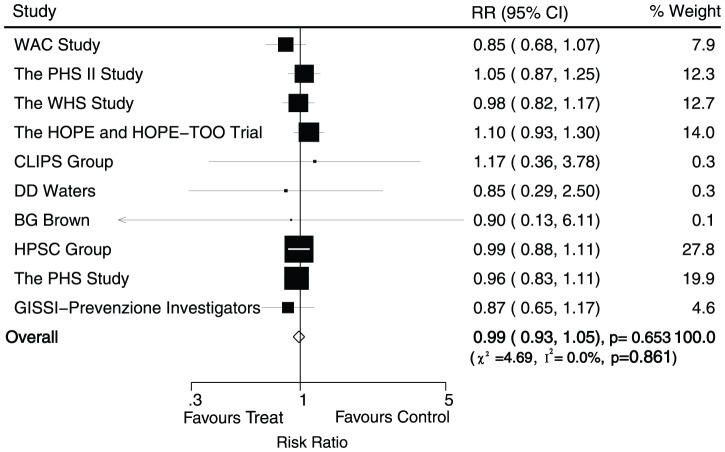
Effects of antioxidant vitamin supplementation on the risk of stroke.

Data for the effect of antioxidant vitamin supplementation on stroke were available from 10 trials, including 127039 individuals and 3749 events of stroke. Overall, the pooled RR showed a 1% reduction in event of stroke, and no evidence showed that antioxidant vitamin supplementation protected against stroke risk (RR, 0.99; 95%CI, 0.93–1.05; P = 0.65, without evidence of heterogeneity, Figure 4)

**Figure 5:**
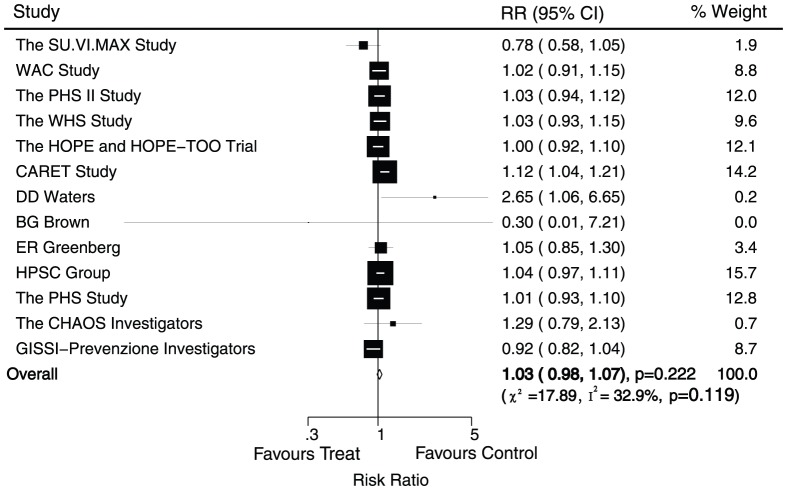
Effects of antioxidant vitamin supplementation on the risk of total death.

Data for the effect of antioxidant vitamin supplementation on total death were available from 13 trials, including 160276 individuals and 14122 events of mortality. No effect of antioxidant vitamin supplementation on the risk of total death was observed (RR, 1.03; 95%CI, 0.98–1.07; P = 0.22, with unimportant heterogeneity, Figure 5). Although heterogeneity was observed in the magnitude of the effect across the trials included, however, after sequential exclusion of each trial from all pooled analysis, the results were not affected by exclusion of any specific trial.

**Figure 6:**
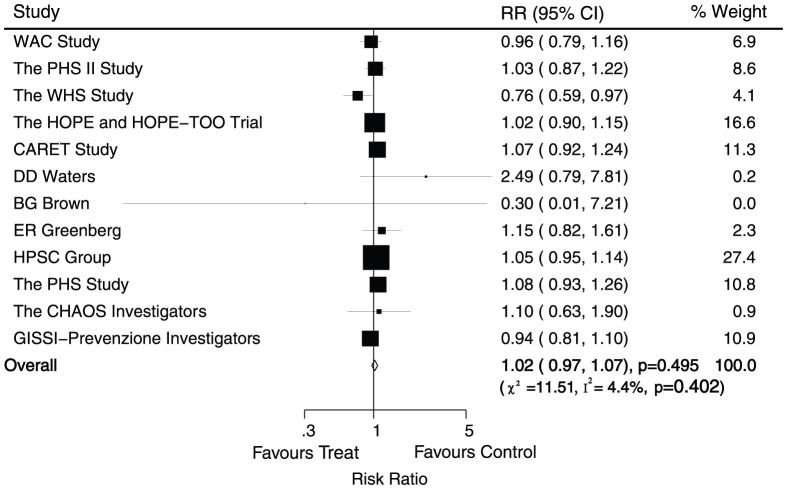
Effects of antioxidant vitamin supplementation on the risk of cardiac death.

Data for the effect of antioxidant vitamin supplementation on cardiac death were available from 12 trials, including 147535 individuals and 5980 events of cardiac death. Overall, there was no evidence to show that antioxidant vitamin supplementation protected against cardiac death (RR, 1.02; 95%CI, 0.97–1.07; P = 0.50, without evidence of heterogeneity, Figure 6).

Four of the trials included 52969 individuals with 6526 revascularization events. No evidence indicated that antioxidant supplementation protected against revascularization risk (RR, 1.00; 95%CI, 0.95–1.05; P = 0.97, with unimportant heterogeneity, Table 2).

**Table 2:** Summary of the odds ratios of all toxicities outcomes assessed.

Outcomes	Included studies	OR and 95% CI	P value	Heterogeneity(%)	P value for heterogeneity
Major cardiovascular events	12	1.00 (0.96–1.03)	0.79	0	0.59
Myocardial infarction	12	0.98 (0.92–1.04)	0.54	28	0.17
Stroke	10	0.99 (0.93–1.05)	0.65	0	0.86
Total death	13	1.03 (0.98–1.07)	0.22	33	0.12
Cardiac death	12	1.02 (0.97–1.07)	0.50	4	0.40
Revascularization	4	1.00 (0.95–1.05)	0.97	18	0.30
Total CHD	2	0.96 (0.87–1.05)	0.35	0	0.78
Angina	2	0.98 (0.90–1.07)	0.66	34	0.22
Congestive heart failure	3	1.07 (0.96–1.19)	0.21	15	0.31

Two trials reported data for the incidence of coronary heart disease, including 35442 individuals and 1906 events of coronary heart disease. Overall, reduction in the risk of coronary heart disease with antioxidant supplementation was not statistically significant (RR, 0.96; 95%CI, 0.87–1.05; P = 0.35, without evidence of heterogeneity, Table 2).

The risk of angina was reported in 2 trials, including 24182 individuals and 2893 events of angina. Overall, the pooled analysis showed no significant differences between antioxidant vitamin supplementation and placebo for angina (RR, 0.98; 95%CI, 0.90–1.07; P = 0.66, with moderate heterogeneity: 34% and P = 0.22, Table 2).

Of the 15 trials included in our study, only 3 provided data about congestive heart failure, which included 24548 individuals and 1811 events of congestive heart failure. Overall, there was no evidence to show that antioxidant vitamin supplementation could reduce the risk of congestive heart failure (RR, 1.07; 95%CI, 0.96 to 1.19; P = 0.21, with unimportant heterogeneity, Table 2).

### Subgroup Analysis

Subgroup analyses were conducted for major cardiovascular events, myocardial infarction, and stroke to evaluate the effect of antioxidant vitamin on cardiovascular outcomes in some specific population. However, no significant differences were detected between the effects of antioxidant vitamin supplementation on major cardiovascular events, myocardial infarction, and stroke based on pre-defined subset factors (Table 3).

**Table 3:** Subgroup analysis for the effect of antioxidant vitamin on major cardiovascular events, myocardial infarction, and stroke.

Outcomes	Group	Relative risk (RR)	P value	heterogeneity (%)	P value for heterogeneity
**Major cardiovascular events**					
	**Publication year**				
	Before 2000	0.99 (0.94–1.04)	0.69	0	0.97
	After 2000	1.00 (0.95–1.05)	0.88	14	0.32
	**Number of patients**				
	≥10000	0.99 (0.95–1.03)	0.59	0	0.97
	<10000	1.01 (0.87–1.17)	0.88	48	0.10
	**Mean age**				
	≥60	1.01 (0.93–1.09)	0.89	29	0.23
	<60	0.97 (0.91–1.03)	0.32	0	0.63
	**Sex**				
	Men	0.99 (0.94–1.05)	0.80	0	1.00
	Women	0.94 (0.84–1.06)	0.33	30	0.24
	Both men and women	1.02 (0.96–1.08)	0.58	0	0.42
	**History of disease**				
	Health	0.97 (0.93–1.02)	0.26	0	0.88
	High risk for cardiovascular events	1.02 (0.96–1.08)	0.49	13	0.33
	**Duration of follow-up (year)**				
	≥6	0.99 (0.95–1.03)	0.66	0	0.69
	<6	1.01 (0.92–1.12)	0.83	25	0.26
	**Jadad score**				
	4 or 5	0.99 (0.95–1.04)	0.78	6	0.39
	<4	1.00 (0.93–1.06)	0.89	0	0.64
**Myocardial infarction**					
	**Publication year**				
	Before 2000	0.95 (0.84–1.08)	0.45	61	0.06
	After 2000	1.00 (0.93–1.07)	0.96	6	0.38
	**Number of patients**				
	≥10000	0.98 (0.93–1.04)	0.53	0	0.82
	<10000	0.90 (0.68–1.18)	0.43	61	0.03
	**Mean age**				
	≥60	0.92 (0.77–1.10)	0.35	61	0.03
	<60	1.01 (0.93–1.10)	0.82	0	0.58
	**Sex**				
	Men	0.97 (0.89–1.05)	0.42	0	0.39
	Women	0.97 (0.83–1.12)	0.67	0	0.84
	Both men and women	0.94 (0.77–1.16)	0.59	67	0.02
	**History of disease**				
	Health	0.97 (0.90–1.04)	0.36	0	0.69
	High risk for cardiovascular events	0.98 (0.86–1.11)	0.74	51	0.06
	**Duration of follow-up (year)**				
	≥6	1.00 (0.94–1.06)	0.96	0	0.48
	<6	0.92 (0.76–1.12)	0.42	53	0.06
	**Jadad score**				
	4 or 5	1.00 (0.94–1.06)	0.98	0	0.58
	<4	0.93 (0.75–1.14)	0.47	68	0.02
**Stroke**					
	**Publication year**				
	Before 2000	0.94 (0.83–1.07)	0.37	0	0.57
	After 2000	1.00 (0.93–1.07)	0.99	0	0.81
	**Number of patients**				
	≥10000	0.98 (0.91–1.05)	0.59	0	0.88
	<10000	1.00 (0.88–1.14)	0.96	0	0.49
	**Mean age**				
	≥60	1.02 (0.92–1.13)	0.74	0	0.47
	<60	0.95 (0.82–1.10)	0.50	0	0.81
	**Sex**				
	Men	0.99 (0.89–1.11)	0.89	0	0.47
	Women	0.93 (0.81–1.06)	0.28	0	0.62
	Both men and women	1.04 (0.90–1.20)	0.58	0	0.60
	**History of disease**				
	Health	0.97 (0.89–1.05)	0.45	0	0.57
	High risk for cardiovascular events	1.01 (0.92–1.10)	0.88	0	0.88
	**Duration of follow-up (year)**				
	≥6	0.99 (0.92–1.07)	0.87	0	0.43
	<6	0.97 (0.87–1.08)	0.58	0	0.95
	Jadad score				
	4 or 5	0.99 (0.92–1.06)	0.81	0	0.73
	<4	0.96 (0.84–1.11)	0.60	0	0.74

### Publication Bias

We used Egger’s test [22] and Begg-Mazumdar test [23] to check for potential publication bias, which showed no evidence of publication for the outcomes of major cardiovascular events (P value for Egger’s test, 0.861; P value for Begg-Mazumdar test, 0.115), myocardial infarction (P value for Egger’s test, 0.194; P value for Begg-Mazumdar test, 0.193), stroke (P value for Egger’s test, 0.638; P value for Begg-Mazumdar test, 0.721), total death (P value for Egger’s test, 0.908; P value for Begg-Mazumdar test, 0.760), and cardiac death (P value for Egger’s test, 0.911; P value for Begg-Mazumdar test, 0.945).

## Discussion

This large quantitative meta-analysis included 188209 individuals in fifteen trials with a broad range of baseline characteristics. The results of our study suggested that antioxidant vitamin supplementation does not effect on major cardiovascular events, myocardial infarction, stroke, total death, cardiac death, revascularization, coronary heart disease, angina, and congestive heart failure. Subgroup analyses also supported these conclusions.

There were no significant differences between antioxidant vitamin supplementation and placebo in the relative risk for major cardiovascular outcomes. The reason for this absence difference could be that although LDL susceptibility to oxidative damage could led to atherosclerosis, but the impact of plasma antioxidant vitamin level on vascular events is not primarily related to the pathogenesis of major cardiovascular events, which is largely attributable to plaque formation and rupture. Furthermore, although several trials suggested that different source of antioxidant vitamin contributed a different role on cardiovascular events, however, neither form of antioxidant vitamin appears more or less to be associated with the observation that both antioxidant vitamin sources have similar antioxidant properties. In addition, we noted that antioxidant vitamin does not effects on revascularization, coronary heart disease, angina, and congestive heart failure, which could be that less trial provided the data of these information.

According to the Women’s Health Study [26]–[28], vitamin E provided no overall benefit for major cardiovascular events, total mortality and cardiovascular mortality in healthy women. However, subgroup analysis was observation of a significant 7% reduction in myocardial infarction, and 5% reduction in stroke in patients aged at least 65 years. Furthermore, HOPE trial [29] enrolled individuals aged at least 55 years with cardiovascular risk factors supported that there were no overall effect of antioxidant vitamin on cardiovascular outcomes and no heterogeneity of results by age. Our study were inconsistent with these two trials, and providing the conclusion that antioxidant vitamin does not effect on cardiovascular outcomes when based on age (stratified 60 years old). Recently, the conclusions of randomized controlled trials by Sesso HD et al [49] was similar to our current meta-analysis, and also indicated that multivitamin supplementation did not reduce major cardiovascular events, myocardial infarction, stroke, and cardiac death after more than a decade of treatment and follow-up in US male physicians.

A previous meta-analysis [13] has illustrated that the risk of cardiovascular outcomes is not significantly reduced using vitamin E supplementation compared with placebo. Additionally, beta-carotene led to a small but significant increase in total mortality and cardiac death. Another important review [50] concluded that no evidence to support antioxidant vitamin (beta-carotene, vitamin A, vitamin C, vitamin E, and selenium) supplements for primary or secondary prevention. This conclusion was similar to our current study. In addition, our study provided the additional vitamin C on cardiovascular outcomes. Furthermore, subgroup analyses were carried out based on important factors which could affected the effects of antioxidant vitamin on the risk of cardiovascular outcomes more detailed. In our study, subgroup analysis also supported that antioxidant vitamin supplementation had no effect on cardiovascular outcomes. Furthermore, the initial evidence for a correlation between antioxidant vitamin supplementation and cardiovascular outcomes was provided by observational studies [51], [52], the possibility that these associations merely reflect the effects of other aspects of the diet or lifestyle on cardiovascular disease rates cannot be ruled out, which may led us overestimate the effect of this relationship. Our study is promising because we restricted to randomized controlled trials to meet our inclusion criteria and aimed to provide the best evidence for a causal relationship.

The limitation of this study includes the inherent assumptions made for any meta-analysis, because the analysis uses pooled data either from published papers or provided by individual study authors, individual data and original data were not available, which prevented us doing more detailed relevant analysis and obtaining more comprehensive results. Furthermore, different follow-up times and therapy dose could also affected our conclusions about the association between antioxidant vitamin and major cardiovascular outcomes. Finally, antioxidant vitamin always be considered as combined supplementation, we could not conclude that some are harmful or some are beneficials, intrinsic effects on cardiovascular outcomes might be reduced or balanced by other vitamin or other things in the supplements. Therefore, we just gave a relative result by comparing antioxidant vitamin supplementation with placebo and provided a synthetic and comprehensive review.

In conclusion, the findings of our study indicated that antioxidant vitamin supplementation had no significant effects on major cardiovascular events, myocardial infarction, stroke, total death, cardiac death, and any possible cardiac-related events. In future research, it is important to focus on healthy individuals for primary prevention of cardiovascular disease, and to combine other antioxidant vitamin to provide an optimal strategy. We suggest that the ongoing trials be improved in the following ways: the adverse effects in trials should be recorded and reported normatively, so that the side-effects of any treatment can be evaluated in future trials, the role of treatment duration and dosage should be investigated in more detail to explore optimal dose and duration of treatment.

## Supporting Information

Table S1 **PRISMA Checklist.**
(DOC)

Figure S1 **PRISMA Flowchart.**
(DOC)
