# Supplementary figures and images for: Effect of Antioxidant Vitamin Supplementation on Cardiovascular Outcomes: A Meta-Analysis of Randomized Controlled Trials
Source: PLoS One. 2013 Feb 20;8(2):e56803. doi: 10.1371/journal.pone.0056803 (PMC3577664; doi:10.1371/journal.pone.0056803)

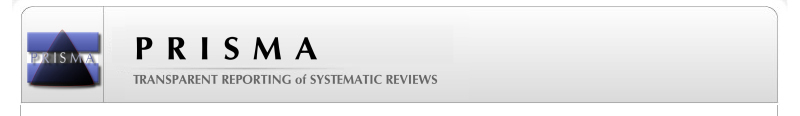
**PRISMA 2009 Flow Diagram**

**Screening**

**Included**

**Eligibility**

**Identification**

Supplement: Figure S1 — PRISMA Flowchart. (DOC) [file pone.0056803.s002.doc]
